# Supplementary material for: Perception and reasons for the choice of informal provider among women receiving antenatal care services from traditional birth attendants in rural communities of Lagos state, Nigeria
Source: PLoS One. 2024 Jun 13;19(6):e0304856. doi: 10.1371/journal.pone.0304856 (PMC11175510; doi:10.1371/journal.pone.0304856)
Supplement: Attachment — Submitted filename: Review comments.docx [file pone.0304856.s002.docx]

**Review comments**

**General comments**

The Authors are commended for conceptualizing this study and their intention to publish their research work. After going through the manuscript, I am of the opinion that there are certain aspects of the study that should be clarified

1. Concept/Title: The study was carried out among women attending ‘antenatal care’ from traditional birth attendants. The title of the manuscript did not bring this into focus. I wonder why assessing the perception of pregnant women on utilization of traditional birth attendants should be carried out among women already utilizing that service. I would have preferred such a concept to be a community based study. In line with the concept of the study, Authors should justify why this study is necessary.
2. Outcome variable: The outcome variable(s) of the study are not well defined. The rationale for using <70% of scores of respondents to categorize perception of respondents is not clear. Again, one wonders the relevance of pattern of utilization as determined in this study to current and future studies involving traditional birth attendants and to the body of knowledge generally. Authors should justify why that outcome variable is of interest.
3. Authors utilized data involving the spouses of the respondents who are less than the sample size of 347 in logistic regression analysis. This is wrong and should be reviewed. The entire results of the logistic regression analysis should be reviewed.
4. Authors should pay attention to guidelines of the Journal in presenting this manuscript especially the abstract section. Also, in presenting the logistic regression analysis in the abstract, Authors should use adjusted odd ratio and 95% confidence interval.
5. The background of the study is well written and Authors are commended for their great efforts.

The responses to the comments above will actually determine the merit of the manuscript in proceeding with the review process.
