# Supplementary material for: Perception and reasons for the choice of informal provider among women receiving antenatal care services from traditional birth attendants in rural communities of Lagos state, Nigeria
Source: PLoS One. 2024 Jun 13;19(6):e0304856. doi: 10.1371/journal.pone.0304856 (PMC11175510; doi:10.1371/journal.pone.0304856)
Supplement: Attachment — Submitted filename: Review comments.docx [file pone.0304856.s003.docx]

**Review comments**

**General comments**

I commend the efforts of the Authors in reviewing this manuscript. I am also of the opinion that certain aspects of the manuscript need to undergo further revision.

Authors should take note of the following observations.

1. **Title of study:** I suggest that the Authors should consider the suggestion below

**Perception and reasons for choice of informal provider among women receiving antenatal care from traditional birth attendants in rural communities of Lagos state, Nigeria**

2. **Abstract**

The acronym TBA was not introduced in the abstract.

Define the outcome variable, ‘utilization’ in the methods section so the result of regression analysis will be meaningful.

Include also in the methods section, how positive perception was determined.

Use; Predictors of use of TBA only included:

3. **Methods**

**Study population**

Line 94; …….., attending TBAs. This should be reviewed.

Line 99, p of 25.5% is a proportion and not a number

4. **Data analysis**

Use the new name for SPSS, Statistical Product and Service Solutions

What was the rationale for using scores <70% as having a positive perception. Any justification? Any reference?

Authors should also take another look at the scoring system for the variables. For example, TBAs can perform Caesarian section.

Utilization as an outcome variable was not explained in the Methods section.

Authors should use variables with p values <0.2 at bivariate analysis as basis for inclusion of variables into the binary logistic regression model.

Refer to, Maldonado G, Greenland S. Simulation study of confounder-selection strategies. Am J Epidemiol. 1993;138:923-36.

5. **Results**

**Socio-demographic characteristics**

Line 135, The proportion 51.9% cannot be regarded as ‘about half’.

**Table 1**

Use Percent instead of Percentage

Include (n=317) for variables related to the spouses

6. **Discussion**

Page 317, should be use of TBA only

Page 318; Those with secondary education and lower used TBA only compared with those with post secondary education. This statement should be reviewed.

There is no need to discuss results of bivariate analysis when that of multivariate analysis are included in the same study.

Line 352; This study was community based with…….. This statement is not correct. The study is like a ‘health facility based’ study and not a community based study. The study took place among those assessing care among traditional birth attendants.

7. **Limitation**

Delete the first statement under this sub-heading. Keep the second statement. Recall the 100% positive perception of the respondents as obtained in this study and state that it may be due to the study being conducted among those assessing care among TBAs.

8. **References**

Authors should ensure that referencing style conforms to Journal guidelines.
