# Supplementary material for: Perception and reasons for the choice of informal provider among women receiving antenatal care services from traditional birth attendants in rural communities of Lagos state, Nigeria
Source: PLoS One. 2024 Jun 13;19(6):e0304856. doi: 10.1371/journal.pone.0304856 (PMC11175510; doi:10.1371/journal.pone.0304856)
Supplement: Attachment — Submitted filename: Response to Reviewers.docx [file pone.0304856.s004.docx]

Response to Reviewers

PONE-D-23-19072R1	05-12-2023

**Perception and reasons for choice of informal provider among women** **receiving antenatal care from traditional birth attendants in rural communities of Lagos state, Nigeria**

Reviewer #2: Review comments

| Reviewers’ comments | Authors’ comments | Page | Line Number |
| --- | --- | --- | --- |
| Title I suggest that the Authors should consider the suggestion below  Perception and reasons for choice of informal provider among women receiving antenatal care from traditional birth attendants in rural communities of Lagos state, Nigeria | The title of the manuscript has been revised accordingly  “Perception and reasons for choice of informal provider among women receiving antenatal care from traditional birth attendants in rural communities of Lagos state, Nigeria” | 1 | Line 1 - 2 |
| Abstract The acronym TBA was not introduced in the abstract.  Define the outcome variable, ‘utilization’ in the methods section so the result of regression analysis will be meaningful.    Include also in the methods section, how positive perception was determined.  Use; Predictors of use of TBA only included: | This has been introduced  Done  Done | 2  5-6  6 | Line 28 – 29  Line 115 – 120  Line 28 – 29 |
| Methods Study population  Line 94; …….., attending TBAs. This should be reviewed.  Line 99, p of 25.5% is a proportion and not a number | This has been adjusted accordingly | 5  5 | Line 94-95  Line 98 |
| Data analysis  Use the new name for SPSS, Statistical Product and Service Solutions    What was the rationale for using scores <70% as having a positive perception. Any justification? Any reference?  Authors should also take another look at the scoring system for the variables. For example, TBAs can perform Caesarian section.    Utilization as an outcome variable was not explained in the Methods section. | Adjusted appropriately “Statistical Product and Service Solutions”  The choice of using scores below 70%, to interpret Likert scale responses as having a positive perception is a subjective decision made by the researchers based on our judgment of the specific study context.  Moreover, we consulted experts in the field to determine an appropriate threshold for interpreting the Likert scale responses.  the correct option here was strongly disagree. This was intentionally included to assess the perception of participants.  This is now explained in the methods section | 5  5-6  6 | Line 113  Line 115-120  Line 121-123 |
| Results  Socio-demographic characteristics  Line 135, The proportion 51.9% cannot be regarded as ‘about half’.  Table 1  Use Percent instead of Percentage  Include (n=317) for variables related to the spouses | Corrected  Done.  NB: Variables related to spouses is 337 not 317 | 6  7 | Line 136-137  Line 143-144 |
| Discussion  Page 317, should be use of TBA only  Page 318; Those with secondary education and lower used TBA only compared with those with post secondary education. This statement should be reviewed. | Done  Respondents’ level of education was significantly associated with the use of TBA only.  Reviewed.  Those who had lower up to secondary education levels used only TBAs compared with those with post-secondary education for ANC. | 18  18 | Line 319  Line 319-321 |
| Line 352; This study was community based with…….. This statement is not correct. The study is like a ‘health facility based’ study and not a community based study. The study took place among those assessing care among traditional birth attendants. | Noted. The statement has been deleted |  |  |
| Limitation  Delete the first statement under this sub-heading. Keep the second statement. Recall the 100% positive perception of the respondents as obtained in this study and state that it may be due to the study being conducted among those assessing care among TBAs. | DONE; Rephrased as  “Causation cannot be ascribed to the relationships found in this study. The 100% positive perception of the respondents obtained in this study may be due to the study being conducted among pregnant women assessing care with TBAs.” | 20 | Line 357-359 |
| References  Authors should ensure that referencing style conforms to Journal guidelines. | This is ensured. Thank you |  |  |
