# Supplementary material for: Perception and reasons for the choice of informal provider among women receiving antenatal care services from traditional birth attendants in rural communities of Lagos state, Nigeria
Source: PLoS One. 2024 Jun 13;19(6):e0304856. doi: 10.1371/journal.pone.0304856 (PMC11175510; doi:10.1371/journal.pone.0304856)
Supplement: Attachment — Submitted filename: Response to Reviewer.docx [file pone.0304856.s005.docx]

Authors should take note of the following points;
1. Basis for inclusion of variables into the logistic regression model after bivariate analysis. This was not stated all through the manuscript but from the results of the study as presented, Authors only included variables that were statistically significant at p<0.05. This may not be the best approach. I encourage Authors to use a cut off p value of <0.2 for inclusion of variables into the regression model after bivariate analysis. Refer to

Maldonado G, Greenland S. Simulation study of confounder-selection strategies. American Journal of Epidemiology. 1993;138(11).

RESPONSE: We have now applied a cut-off p-value of <0.2 for the inclusion of variables into the regression model after bivariate analysis. (Pg. 6; Line 122-123)

The basis for the inclusion of variables into the logistic regression model after bivariate analysis have been stated. . (Pg. 6; Line 117-122)

2. The presentation of the Abstract was not done according to Journal guidelines. Authors should refer to the website of the Journal for guidance.

RESPONSE: The abstract is now presented according to the journal guidelines with sections. (Pg. 2-3; Line 29-56)

Also, positive perception and utilization being the outcome variables ought to be defined in the methods section of the abstract.

RESPONSE:

Positive perception of TBA services corresponds with a score of <70%. Utilization of ANC services was dichotomized into the use of TBA alone or the use of a combination of TBA and HCFs for ANC services. This has been included in the manuscript. (Pg. 6; Line 130-132)

Authors should also have the keywords indicated.

RESPONSE: The keywords were included in the appropriate section of PLOS ONE Editorial Manager Submission system (ONLINE)

3. In the Background section, the first definition of TBA should suffice.

RESPONSE: Only the first definition of TBA is now maintained in the manuscript. (Pg. 3; Line 64-66)

4. Authors should indicate the date internet articles were accessed in the reference section. They should also ensure that use of references conform to Journal guidelines.

RESPONSE: DONE. (References)

5. Instead of ‘None’ for education of spouse, Authors should use No formal education and sample size, (n=337) should be placed under the Frequency column for uniformity.

RESPONSE:

These corrections have been effected in the manuscript accordingly. (Pg. 8; Line 159-160)

For table 2, reporting of results should be based on proportions instead of raw figures.

These corrections have been effected in the manuscript accordingly. (Pgs. 8-9; Line 163-173)

6. Provide the rationale for using <70% of the total score as basis for categorizing a respondent as having good perception of TBAs.

RESPONSE: Theoretically, if the median score is used, respondents who have a neutral response for all items will be wrongly classified as having a negative perception (less supportive) of TBAs, hence, the need to increase the cut off point to 70%.
