# Supplementary material for: Perception and reasons for the choice of informal provider among women receiving antenatal care services from traditional birth attendants in rural communities of Lagos state, Nigeria
Source: PLoS One. 2024 Jun 13;19(6):e0304856. doi: 10.1371/journal.pone.0304856 (PMC11175510; doi:10.1371/journal.pone.0304856)
Supplement: Attachment — Submitted filename: Review comments.docx [file pone.0304856.s006.docx]

**Review comments**

**General comment**

I commend the Authors for the review of the manuscript so far. I was of the opinion that this review will be the last however there are some observations I have made which may necessitate another review of the manuscript by the Authors. I plead with the Authors to bear with me in this regard.

Authors should take note of the following points

1. **Table 5: Predictors of health service utilization among the respondents**. Authors included the educational level of the Spouse in the logistic regression model along with other variables. From table 1, there are 347 respondents of which 337 are living with their spouses. This means that the regression was done based on 337 which is the number common to all. This wrong. Authors should repeat the regression analysis without including the educational level of the Spouse. The inclusion of the educational level of the Spouse for further analysis should stop at the bivariate level.
2. Naming the outcome variable, **‘Use of health service utilization’** appear very confusing and may be misunderstood by Readers. The outcome measure should be utilizing only TBA services in current pregnancy. Authors are free to paraphrase the outcome measure as stated.
3. In the abstract, the results of the logistic regression analysis should be represented using adjusted odds ratio and 95% confidence interval. For example, Predictors of utilizing only TBA services in current pregnancy included …………
4. Define ‘positive perception’ in the methods section of abstract.
5. SPSS is currently referred to as **Statistical Product and Service Solutions**. Authors should reflect this correction in the manuscript.
6. Respondents should report the results of the bivariate analysis bearing in mind the difference in proportions. For example, ‘The highest proportion of respondents who utilized only TBA services, 80.9% had primary education while the least, 38.8% have attained tertiary education and the difference in proportions was found to be statistically significant, (**χ^2^ =**39.896, p<0.001)
7. Authors should present the tables in the manuscript in line with Journal guidelines. Authors should refer to the Journal website for guidance.
8. Authors should ‘expand’ the study limitation so as to enhance the understanding of Readers. The caption ‘Limitation’ may be deleted.
9. In table 3, use ‘estimated distance’ instead of ‘distance’. In Line 101, use ‘TBAs’. Line 141, use ‘name’ instead of ‘names’ Line 164, use ‘Less than half of the respondents, 43.6%.
10. Use ‘Percent’ instead of ‘Percentage’. p<0.001 instead of p=<0.001. In table 2 include the meaning of TBA as a foot note.
