# Supplementary material for: Perception and reasons for the choice of informal provider among women receiving antenatal care services from traditional birth attendants in rural communities of Lagos state, Nigeria
Source: PLoS One. 2024 Jun 13;19(6):e0304856. doi: 10.1371/journal.pone.0304856 (PMC11175510; doi:10.1371/journal.pone.0304856)
Supplement: Attachment — Submitted filename: Response to Reviewers comments.docx [file pone.0304856.s007.docx]

**Response to Reviewers**

PONE-D-23-19072R4	30-03-2024

**Perception and reasons for the choice of informal provider among women receiving antenatal care services from traditional birth attendants in rural communities of Lagos state, Nigeria**

Reviewer #2: Review comments

| **S/no** | **Reviewers’ comments** | **Authors’ comments** | **Page** | **Line Number** |
| --- | --- | --- | --- | --- |
| **1.** | **Table 5: Predictors of health service utilization among the respondents**. Authors included the educational level of the Spouse in the logistic regression model along with other variables. From table 1, there are 347 respondents of which 337 are living with their spouses. This means that the regression was done based on 337 which is the number common to all. This wrong. Authors should repeat the regression analysis without including the educational level of the Spouse. The inclusion of the educational level of the Spouse for further analysis should stop at the bivariate level. | We have repeated the regression analysis without including the educational level of the Spouse. The inclusion of the educational level of the Spouse for further analysis should has now stopped at the bivariate level. | 17 | 258-261 |
| 2. | Naming the outcome variable, **‘Use of health service utilization’** appear very confusing and may be misunderstood by Readers. The outcome measure should be utilizing only TBA services in current pregnancy. Authors are free to paraphrase the outcome measure as stated. | The outcome measure has been rephrased as “utilizing only TBA services in current pregnancy”  **Table 3:** Utilization of TBA services only in current pregnancy among respondents  Adjustment has been made throughout the manuscript | 12-13 | 215-216 |
| 3. | In the abstract, the results of the logistic regression analysis should be represented using adjusted odds ratio and 95% confidence interval. For example, Predictors of utilizing only TBA services in current pregnancy included ………… | The results of the logistic regression analysis have been represented using adjusted odds ratio and 95% confidence interval in the abstract | 3 | 54-56 |
| 4. | Define ‘positive perception’ in the methods section of abstract. | Defined in method section. | 2 | 42-43 |
| 5. | SPSS is currently referred to as **Statistical Product and Service Solutions**. Authors should reflect this correction in the manuscript. | corrected in the manuscript | 6 | 121 |
| 6. | Respondents should report the results of the bivariate analysis bearing in mind the difference in proportions. For example, ‘The highest proportion of respondents who utilized only TBA services, 80.9% had primary education while the least, 38.8% have attained tertiary education and the difference in proportions was found to be statistically significant, (**χ^2^ =**39.896, p<0.001) | Report of the results of the bivariate analysis has been reported in proportions. | 13-14 | 219-234 |
| 7. | Authors should present the tables in the manuscript in line with Journal guidelines. Authors should refer to the Journal website for guidance. | Done. Each variables are now in separate cells according to the Journal guidelines | 8-17 |  |
| 8. | Authors should ‘expand’ the study limitation so as to enhance the understanding of Readers. The caption ‘Limitation’ may be deleted. | This has been done. The caption ‘Limitation’ is deleted. | 23 | 398-402 |
| 9. | In table 3, use ‘estimated distance’ instead of ‘distance’. In Line 101, use ‘TBAs’. Line 141, use ‘name’ instead of ‘names’ Line 164, use ‘Less than half of the respondents, 43.6%. | All the corrections has been effected accordingly |  |  |
| 10. | Use ‘Percent’ instead of ‘Percentage’. p<0.001 instead of p=<0.001. In table 2 include the meaning of TBA as a foot note. | Done. meaning of TBA has been included as a foot note in table 2 | 10 | 181-182 |
