# Supplementary material for: Perception and reasons for the choice of informal provider among women receiving antenatal care services from traditional birth attendants in rural communities of Lagos state, Nigeria
Source: PLoS One. 2024 Jun 13;19(6):e0304856. doi: 10.1371/journal.pone.0304856 (PMC11175510; doi:10.1371/journal.pone.0304856)
Supplement: Attachment — Submitted filename: Review comments.docx [file pone.0304856.s008.docx]

**Review comments**

**General comment**

My intention was to approve the publication of this manuscript based on this review. However on close scrutiny, there is a flaw in the analysis and interpretation of study findings which will be too grave to ignore. This may necessitate that the manuscript may require another revision before it could be accepted for publication. I urge the Authors to critically review this manuscript before the next submission.

Authors should take note of the following observations.

1. **Binary logistic regression result.** In the abstract, Authors made it look like having a health insurance coverage predicted the use of TBA only in the study which is the outcome variable. See also Lines 248-250 in the result section and lines 388-390 in the discussion section.

Compare these comments with what was showed in table 4 where a significantly higher proportion of respondents who had no insurance coverage, 76.3% utilized only TBA services when compared with those who had insurance coverage, 41.7% (p<0.001). See also the result in table 5 on the same independent variable. I wonder why there was a re-arrangement of the categories of the independent variable ‘Having insurance coverage’ in tables 4 and 5.

IBM-SPSS by default utilizes the last category of the independent variable as the reference category in logistic regression analysis. The question will be whether the Authors coded the variable, ‘Respondent having insurance coverage’ in two different forms in the data set. This is why the results of Chi square and logistic regression analysis are sometimes included in the same table.

Also, the result as displayed for the educational attainment of the respondents in tables 4 and 5 seem not to move in tandem. This is because at bivariate analysis (Chi square), 58.3% of respondents who had no formal education utilized only TBA services as against 38.8% for those who have attained tertiary education. However, at logistic regression analysis, the respondents who have attained tertiary education were 3.480 times more likely to utilize TBA services only.

Authors should endeavor to clarify these two observations in the next submission and if possible seek the services of a Statistician.

2. Minor corrections. Line 41, use ‘included’ instead of ‘include’.

In line 52, use ‘perceived spiritual protection’. Line 55 should be having no health insurance coverage. Report the finding on educational level accordingly after the re-analysis.

In line 68, use TBAs. Use TBAs also in line 71 and 82.

Line 195 should also be perceived spiritual protection.

Line 231, use higher proportion since the categories of the independent variable are two in number. Delete least in line 233.

In table 5, use p<0.001 instead of 0.000.

Authors should remember to report findings with regards to health insurance and educational level at logistic regression analysis accordingly.
