# Supplementary material for: Perception and reasons for the choice of informal provider among women receiving antenatal care services from traditional birth attendants in rural communities of Lagos state, Nigeria
Source: PLoS One. 2024 Jun 13;19(6):e0304856. doi: 10.1371/journal.pone.0304856 (PMC11175510; doi:10.1371/journal.pone.0304856)
Supplement: Attachment — Submitted filename: Response to Reviewers comments.docx [file pone.0304856.s009.docx]

**Response to Reviewers/Rebuttal letter**

PONE-D-23-19072R5

12-05-2024

**Perception and reasons for the choice of informal provider among women receiving antenatal care services from traditional birth attendants in rural communities of Lagos state, Nigeria**

Reviewer #2: Review comments

| **S/no** | **Reviewers’ comments** | **Authors’ comments** | **Page** | **Line Number** |
| --- | --- | --- | --- | --- |
| **1.** | Binary logistic regression result. In the abstract, Authors made it look like having a health insurance coverage predicted the use of TBA only in the study which is the outcome variable. See also Lines 248-250 in the result section and lines 388-390 in the discussion section.  Compare these comments with what was showed in table 4 where a significantly higher proportion of respondents who had no insurance coverage, 76.3% utilized only TBA services when compared with those who had insurance coverage, 41.7% (p<0.001). See also the result in table 5 on the same independent variable. I wonder why there was a re-arrangement of the categories of the independent variable ‘Having insurance coverage’ in tables 4 and 5. | Thank you so much sir, for taking note of this. It was a great mistake due to a re-arrangement of the categories of the independent variable. This has been corrected a new table 5 which was re-run by a statistician has been inserted with necessary adjustments accordingly. | 15 | 241-242 |
| 2. | 2. Minor corrections. Line 41, use ‘included’ instead of ‘include’.  In line 52, use ‘perceived spiritual protection’.  In line 68, use TBAs. Use TBAs also in line 71 and 82.  Line 195 should also be perceived spiritual protection.  Line 231, use higher proportion since the categories of the independent variable are two in number. Delete least in line 233.  In table 5, use p<0.001 instead of 0.000. | All has been effected in their respectives lines as indicated. | As specified | |
| 3. | Authors should remember to report findings with regards to health insurance and educational level at logistic regression analysis accordingly. |  |  | |
| 4. | Reference 39 is OFF | REMOVED AND DELETED BECAUSE TABLE 5 HAS BEEN REVISED. The reference 39 is no longer useful | | |
